# Illinois State Dental Society

**Published:** 1891-05

**Authors:** Louis Ottofy

**Affiliations:** Sec'y.


### ILLINOIS STATE DENTAL SOCIETY.

At the twenty-seventh annual meeting of the Illinois State Dental Society, held at Bloomington, May 12 to 15, 1891, the following officers were elected for the ensuing year: President, W. H. Taggart, Freeport; Vice-President, Garrett Newkirk, Chicago; Secretary, Louis Ottofy, Chicago; Treasurer, W. A. Stevens, Chicago; Librarian, F. H. McIntosh, Bloomington.

The next annual meeting will be held, beginning on the second Tuesday in May, 1892, at Springfield.

LOUIS OTTOFY, *Sec'y.*

